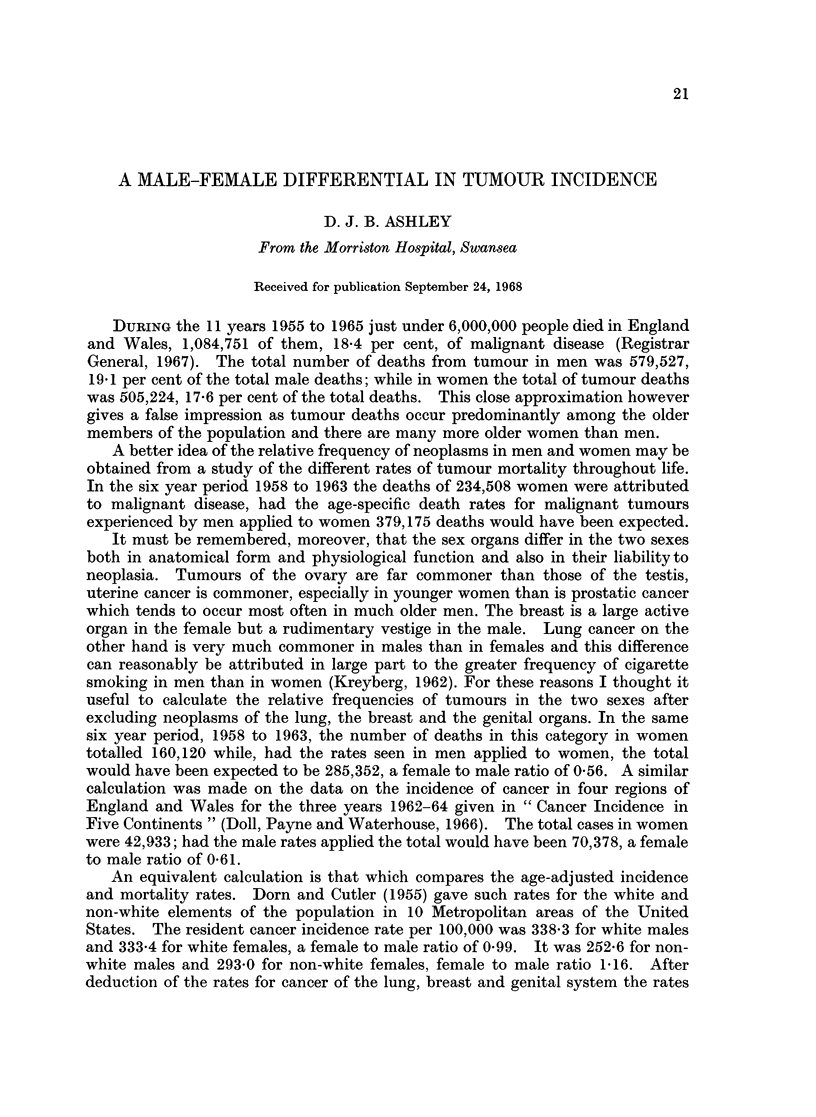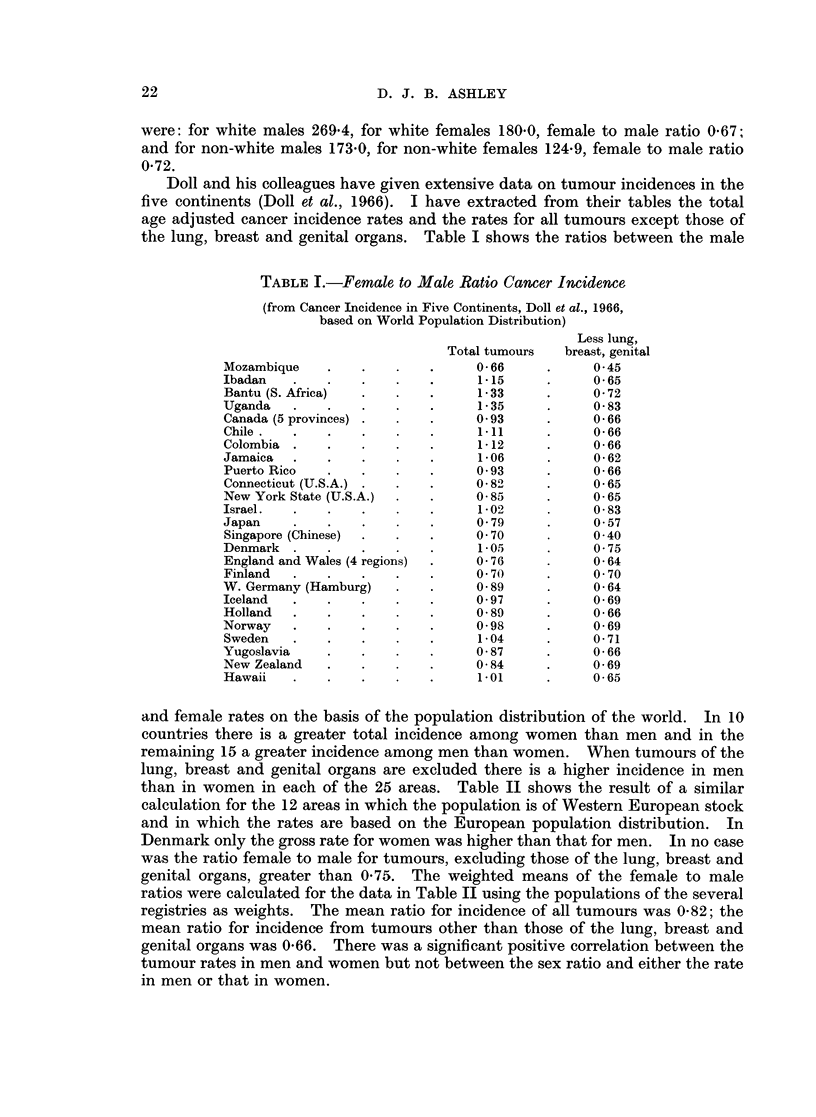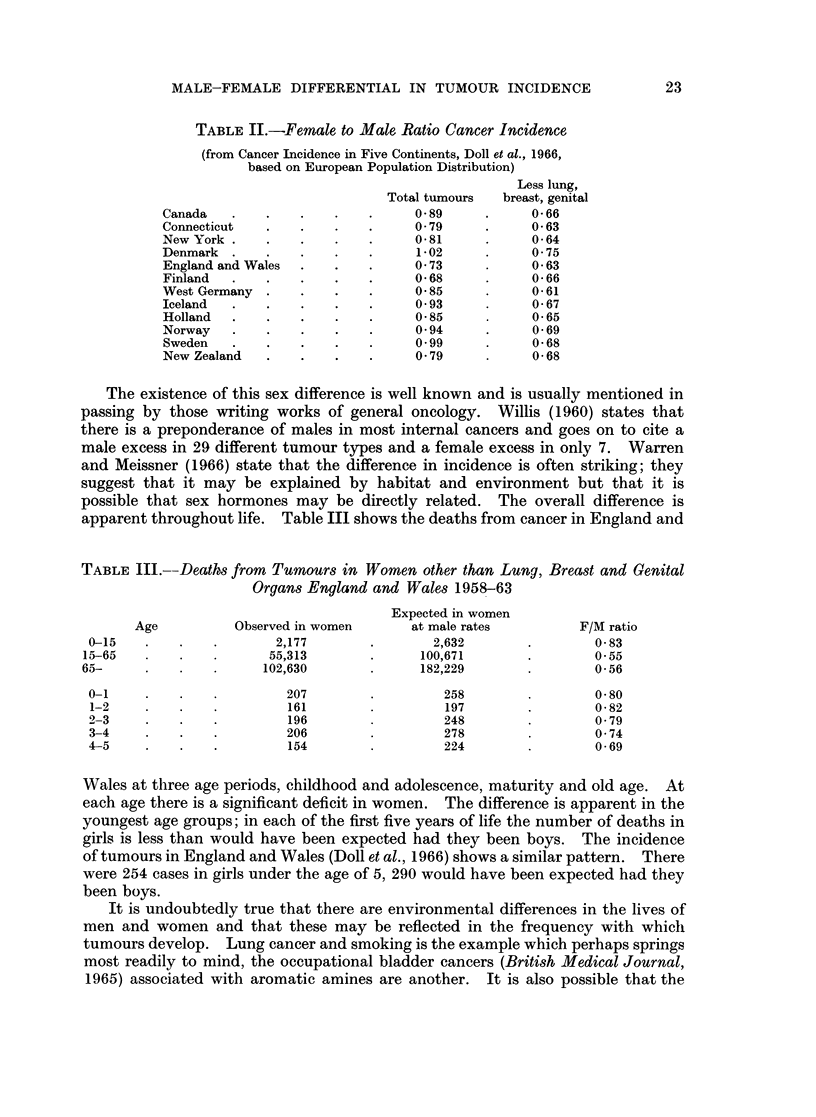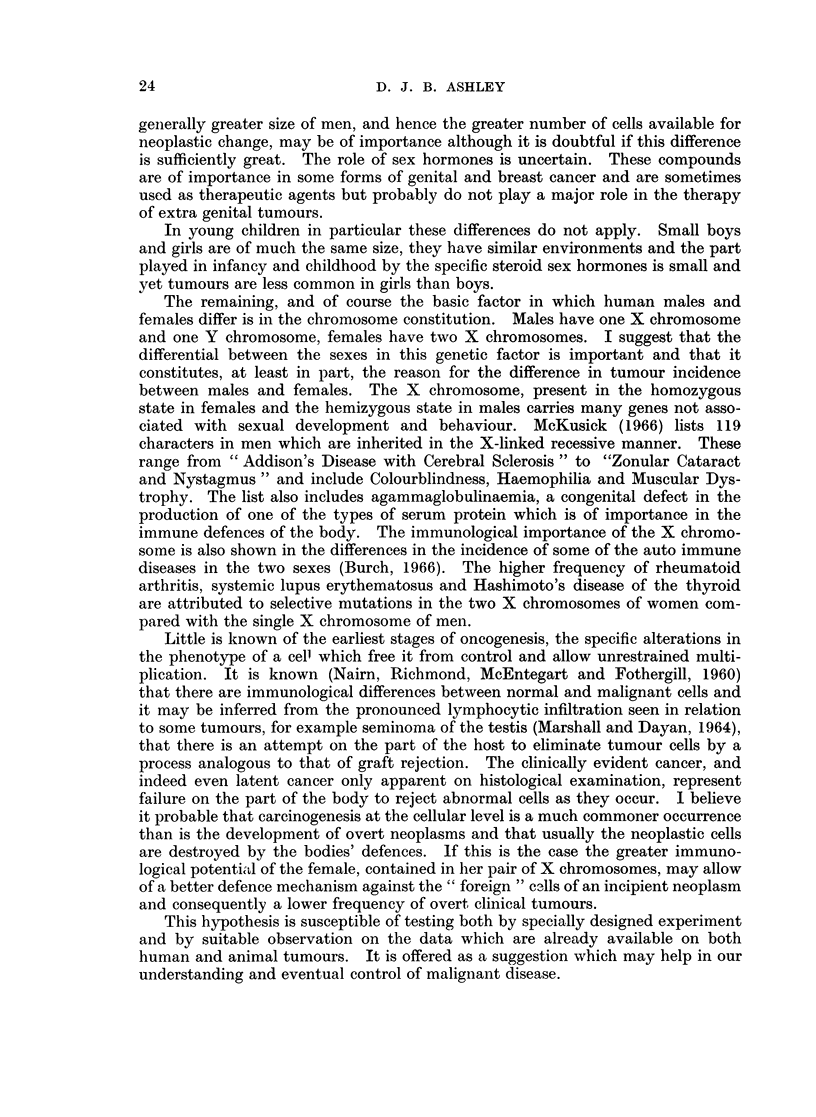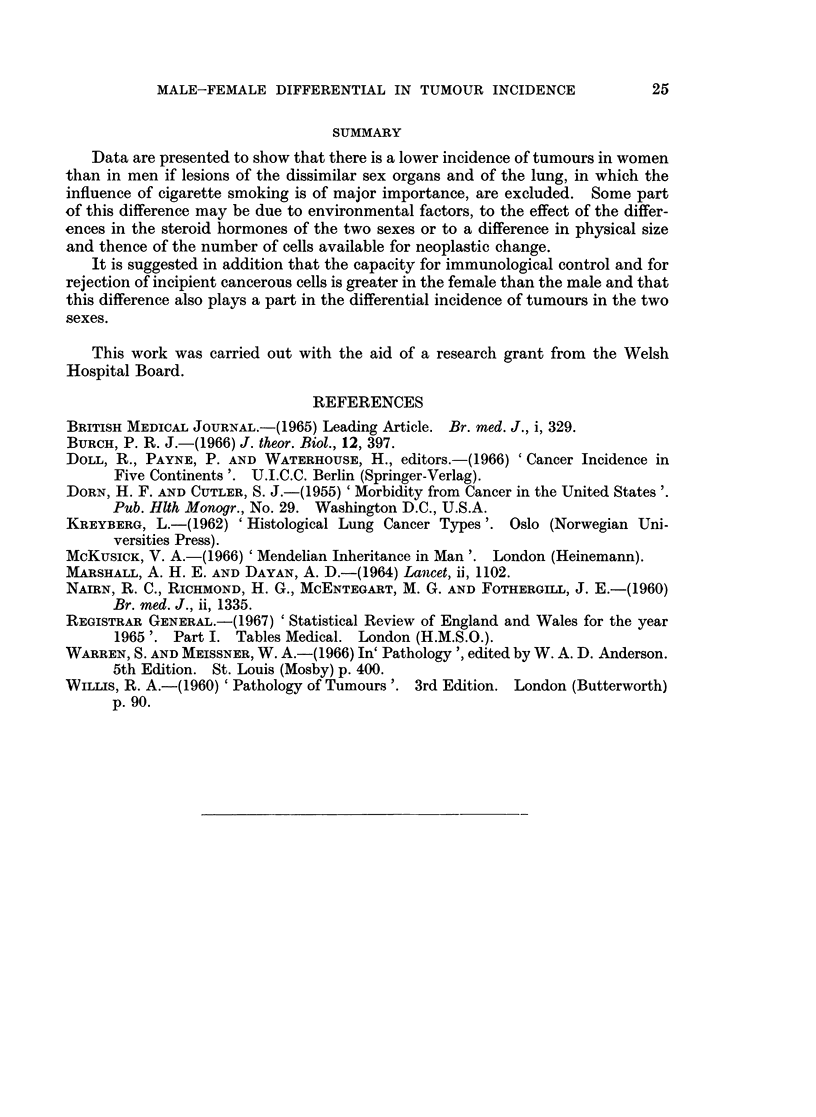# A male-female differential in tumour incidence.

**DOI:** 10.1038/bjc.1969.3

**Published:** 1969-03

**Authors:** D. J. Ashley


### A MALE-FEMALE DIFFERENTIAL IN TUMOUR INCIDENCE

D. J. B. ASHLEY

*From the Morriston Hospital, Swansea*

Received for publication September 24, 1968

DURING the 11 years 1955 to 1965 just under 6,000,000 people died in England and Wales, 1,084,751 of them, 18·4 per cent, of malignant disease (Registrar General, 1967). The total number of deaths from tumour in men was 579,527, 19·1 per cent of the total male deaths; while in women the total of tumour deaths was 505,224, 17·6 per cent of the total deaths. This close approximation however gives a false impression as tumour deaths occur predominantly among the older members of the population and there are many more older women than men.

A better idea of the relative frequency of neoplasms in men and women may be obtained from a study of the different rates of tumour mortality throughout life. In the six year period 1958 to 1963 the deaths of 234,508 women were attributed to malignant disease, had the age-specific death rates for malignant tumours experienced by men applied to women 379,175 deaths would have been expected.

It must be remembered, moreover, that the sex organs differ in the two sexes both in anatomical form and physiological function and also in their liability to neoplasia. Tumours of the ovary are far commoner than those of the testis, uterine cancer is commoner, especially in younger women than is prostatic cancer which tends to occur most often in much older men. The breast is a large active organ in the female but a rudimentary vestige in the male. Lung cancer on the other hand is very much commoner in males than in females and this difference can reasonably be attributed in large part to the greater frequency of cigarette smoking in men than in women (Kreyberg, 1962). For these reasons I thought it useful to calculate the relative frequencies of tumours in the two sexes after excluding neoplasms of the lung, the breast and the genital organs. In the same six year period, 1958 to 1963, the number of deaths in this category in women totalled 160,120 while, had the rates seen in men applied to women, the total would have been expected to be 285,352, a female to male ratio of 0·56. A similar calculation was made on the data on the incidence of cancer in four regions of England and Wales for the three years 1962–64 given in " Cancer Incidence in Five Continents " (Doll, Payne and Waterhouse, 1966). The total cases in women were 42,933; had the male rates applied the total would have been 70,378, a female to male ratio of 0·61.

An equivalent calculation is that which compares the age-adjusted incidence and mortality rates. Dorn and Cutler (1955) gave such rates for the white and non-white elements of the population in 10 Metropolitan areas of the United States. The resident cancer incidence rate per 100,000 was 338·3 for white males and 333·4 for white females, a female to male ratio of 0·99. It was 252·6 for non-white males and 293·0 for non-white females, female to male ratio 1·16. After deduction of the rates for cancer of the lung, breast and genital system the rates were: for white males 269·4, for white females 180·0, female to male ratio 0·67; and for non-white males 173·0, for non-white females 124·9, female to male ratio 0·72.

Doll and his colleagues have given extensive data on tumour incidences in the five continents (Doll *et al.*, 1966). I have extracted from their tables the total age adjusted cancer incidence rates and the rates for all tumours except those of the lung, breast and genital organs. Table I shows the ratios between the male

TABLE I.—*Female to Male Ratio Cancer Incidence*

(from Cancer Incidence in Five Continents, Doll *et al.*, 1966, based on World Population Distribution)

| | Total tumours | Less lung, breast, genital |
|---|---|---|
| Mozambique | 0·66 | 0·45 |
| Ibadan | 1·15 | 0·65 |
| Bantu (S. Africa) | 1·33 | 0·72 |
| Uganda | 1·35 | 0·83 |
| Canada (5 provinces) | 0·93 | 0·66 |
| Chile | 1·11 | 0·66 |
| Colombia | 1·12 | 0·66 |
| Jamaica | 1·06 | 0·62 |
| Puerto Rico | 0·93 | 0·66 |
| Connecticut (U.S.A.) | 0·82 | 0·65 |
| New York State (U.S.A.) | 0·85 | 0·65 |
| Israel | 1·02 | 0·83 |
| Japan | 0·79 | 0·57 |
| Singapore (Chinese) | 0·70 | 0·40 |
| Denmark | 1·05 | 0·75 |
| England and Wales (4 regions) | 0·76 | 0·64 |
| Finland | 0·70 | 0·70 |
| W. Germany (Hamburg) | 0·89 | 0·64 |
| Iceland | 0·97 | 0·69 |
| Holland | 0·89 | 0·66 |
| Norway | 0·98 | 0·69 |
| Sweden | 1·04 | 0·71 |
| Yugoslavia | 0·87 | 0·66 |
| New Zealand | 0·84 | 0·69 |
| Hawaii | 1·01 | 0·65 |

and female rates on the basis of the population distribution of the world. In 10 countries there is a greater total incidence among women than men and in the remaining 15 a greater incidence among men than women. When tumours of the lung, breast and genital organs are excluded there is a higher incidence in men than in women in each of the 25 areas. Table II shows the result of a similar calculation for the 12 areas in which the population is of Western European stock and in which the rates are based on the European population distribution. In Denmark only the gross rate for women was higher than that for men. In no case was the ratio female to male for tumours, excluding those of the lung, breast and genital organs, greater than 0·75. The weighted means of the female to male ratios were calculated for the data in Table II using the populations of the several registries as weights. The mean ratio for incidence of all tumours was 0·82; the mean ratio for incidence from tumours other than those of the lung, breast and genital organs was 0·66. There was a significant positive correlation between the tumour rates in men and women but not between the sex ratio and either the rate in men or that in women.

TABLE II.—*Female to Male Ratio Cancer Incidence*

(from Cancer Incidence in Five Continents, Doll *et al.*, 1966, based on European Population Distribution)

| | Total tumours | Less lung, breast, genital |
|---|---|---|
| Canada | 0·89 | 0·66 |
| Connecticut | 0·79 | 0·63 |
| New York | 0·81 | 0·64 |
| Denmark | 1·02 | 0·75 |
| England and Wales | 0·73 | 0·63 |
| Finland | 0·68 | 0·66 |
| West Germany | 0·85 | 0·61 |
| Iceland | 0·93 | 0·67 |
| Holland | 0·85 | 0·65 |
| Norway | 0·94 | 0·69 |
| Sweden | 0·99 | 0·68 |
| New Zealand | 0·79 | 0·68 |

The existence of this sex difference is well known and is usually mentioned in passing by those writing works of general oncology. Willis (1960) states that there is a preponderance of males in most internal cancers and goes on to cite a male excess in 29 different tumour types and a female excess in only 7. Warren and Meissner (1966) state that the difference in incidence is often striking; they suggest that it may be explained by habitat and environment but that it is possible that sex hormones may be directly related. The overall difference is apparent throughout life. Table III shows the deaths from cancer in England and

TABLE III.—*Deaths from Tumours in Women other than Lung, Breast and Genital Organs England and Wales* 1958–63

| Age | Observed in women | Expected in women at male rates | F/M ratio |
|---|---|---|---|
| 0–15 | 2,177 | 2,632 | 0·83 |
| 15–65 | 55,313 | 100,671 | 0·55 |
| 65– | 102,630 | 182,229 | 0·56 |
| 0–1 | 207 | 258 | 0·80 |
| 1–2 | 161 | 197 | 0·82 |
| 2–3 | 196 | 248 | 0·79 |
| 3–4 | 206 | 278 | 0·74 |
| 4–5 | 154 | 224 | 0·69 |

Wales at three age periods, childhood and adolescence, maturity and old age. At each age there is a significant deficit in women. The difference is apparent in the youngest age groups; in each of the first five years of life the number of deaths in girls is less than would have been expected had they been boys. The incidence of tumours in England and Wales (Doll *et al.*, 1966) shows a similar pattern. There were 254 cases in girls under the age of 5, 290 would have been expected had they been boys.

It is undoubtedly true that there are environmental differences in the lives of men and women and that these may be reflected in the frequency with which tumours develop. Lung cancer and smoking is the example which perhaps springs most readily to mind, the occupational bladder cancers (*British Medical Journal*, 1965) associated with aromatic amines are another. It is also possible that the generally greater size of men, and hence the greater number of cells available for neoplastic change, may be of importance although it is doubtful if this difference is sufficiently great. The role of sex hormones is uncertain. These compounds are of importance in some forms of genital and breast cancer and are sometimes used as therapeutic agents but probably do not play a major role in the therapy of extra genital tumours.

In young children in particular these differences do not apply. Small boys and girls are of much the same size, they have similar environments and the part played in infancy and childhood by the specific steroid sex hormones is small and yet tumours are less common in girls than boys.

The remaining, and of course the basic factor in which human males and females differ is in the chromosome constitution. Males have one X chromosome and one Y chromosome, females have two X chromosomes. I suggest that the differential between the sexes in this genetic factor is important and that it constitutes, at least in part, the reason for the difference in tumour incidence between males and females. The X chromosome, present in the homozygous state in females and the hemizygous state in males carries many genes not associated with sexual development and behaviour. McKusick (1966) lists 119 characters in men which are inherited in the X-linked recessive manner. These range from " Addison's Disease with Cerebral Sclerosis " to "Zonular Cataract and Nystagmus " and include Colourblindness, Haemophilia and Muscular Dystrophy. The list also includes agammaglobulinaemia, a congenital defect in the production of one of the types of serum protein which is of importance in the immune defences of the body. The immunological importance of the X chromosome is also shown in the differences in the incidence of some of the auto immune diseases in the two sexes (Burch, 1966). The higher frequency of rheumatoid arthritis, systemic lupus erythematosus and Hashimoto's disease of the thyroid are attributed to selective mutations in the two X chromosomes of women compared with the single X chromosome of men.

Little is known of the earliest stages of oncogenesis, the specific alterations in the phenotype of a cell which free it from control and allow unrestrained multiplication. It is known (Nairn, Richmond, McEntegart and Fothergill, 1960) that there are immunological differences between normal and malignant cells and it may be inferred from the pronounced lymphocytic infiltration seen in relation to some tumours, for example seminoma of the testis (Marshall and Dayan, 1964), that there is an attempt on the part of the host to eliminate tumour cells by a process analogous to that of graft rejection. The clinically evident cancer, and indeed even latent cancer only apparent on histological examination, represent failure on the part of the body to reject abnormal cells as they occur. I believe it probable that carcinogenesis at the cellular level is a much commoner occurrence than is the development of overt neoplasms and that usually the neoplastic cells are destroyed by the bodies' defences. If this is the case the greater immunological potential of the female, contained in her pair of X chromosomes, may allow of a better defence mechanism against the " foreign " cells of an incipient neoplasm and consequently a lower frequency of overt clinical tumours.

This hypothesis is susceptible of testing both by specially designed experiment and by suitable observation on the data which are already available on both human and animal tumours. It is offered as a suggestion which may help in our understanding and eventual control of malignant disease.

SUMMARY

Data are presented to show that there is a lower incidence of tumours in women than in men if lesions of the dissimilar sex organs and of the lung, in which the influence of cigarette smoking is of major importance, are excluded. Some part of this difference may be due to environmental factors, to the effect of the differences in the steroid hormones of the two sexes or to a difference in physical size and thence of the number of cells available for neoplastic change.

It is suggested in addition that the capacity for immunological control and for rejection of incipient cancerous cells is greater in the female than the male and that this difference also plays a part in the differential incidence of tumours in the two sexes.

This work was carried out with the aid of a research grant from the Welsh Hospital Board.

### REFERENCES

British Medical Journal.—(1965) Leading Article. *Br. med. J.*, i, 329.

Burch, P. R. J.—(1966) *J. theor. Biol.*, **12**, 397.

Doll, R., Payne, P. and Waterhouse, H., editors.—(1966) ' Cancer Incidence in Five Continents '. U.I.C.C. Berlin (Springer-Verlag).

Dorn, H. F. and Cutler, S. J.—(1955) ' Morbidity from Cancer in the United States '. *Pub. Hlth Monogr.*, No. 29. Washington D.C., U.S.A.

Kreyberg, L.—(1962) ' Histological Lung Cancer Types '. Oslo (Norwegian Universities Press).

McKusick, V. A.—(1966) ' Mendelian Inheritance in Man '. London (Heinemann).

Marshall, A. H. E. and Dayan, A. D.—(1964) *Lancet*, ii, 1102.

Nairn, R. C., Richmond, H. G., McEntegart, M. G. and Fothergill, J. E.—(1960) *Br. med. J.*, ii, 1335.

Registrar General.—(1967) ' Statistical Review of England and Wales for the year 1965 '. Part I. Tables Medical. London (H.M.S.O.).

Warren, S. and Meissner, W. A.—(1966) In' Pathology ', edited by W. A. D. Anderson. 5th Edition. St. Louis (Mosby) p. 400.

Willis, R. A.—(1960) ' Pathology of Tumours '. 3rd Edition. London (Butterworth) p. 90.